# The complete chloroplast genome sequence of *Quercus gilva*（Fagaceae）

**DOI:** 10.1080/23802359.2019.1637299

**Published:** 2019-07-12

**Authors:** Qin-Meng Zeng, Bin Liu, Ru-Qiang Lin, Yu-Ting Jiang, Zhong-Jian Liu, Shi-Pin Chen

**Affiliations:** a College of Forestry, Fujian Agriculture and Forestry University, Fuzhou, China;; b Camellia Oleifera Research Center at College of Forestry, Fujian Agriculture and Forestry University, Fuzhou, China;; c Key Laboratory of National Forestry and Grassland Administration for Orchid Conservation and Utilization at College of Forestry, Fujian Agriculture and Forestry University, Fuzhou, China;; d College of Landscape, Fujian Agriculture and Forestry University, Fuzhou, China

**Keywords:** *Quercus gilva*, plastid genome, phylogeny, Fagaceae

## Abstract

*Quercus gilva* is classified as the national second-class protective tree species in China and is widely distributed in Middle East Asia. We determined the complete chloroplast genome sequence for *Q. gilva* using Illumina sequencing data. The complete chloroplast sequence is 160,763 bp, including large single-copy (LSC) region of 90,292 bp, small single-copy (SSC) region of 18,831 bp, and a pair of invert repeats (IR) regions of 25,820 bp. Plastid genome contains 128 genes, 80 protein-coding genes, 40 tRNA genes, and 8 rRNA genes. Phylogenetic analysis based on 26 chloroplast genomes indicates that *Q. gilva* is closely related to *Q. sichourensis* in Fagaceae.

*Quercus gilva* belongs to Fagaceae, it is widely distributed in Middle East Asia. It is one of the main tree species in the distribution area. *Quercus gilva* grows at an altitude of 300 m to 1500 m above sea level in mountainous areas (Luo and Xu 1995). The tree species has the advantages of strong-adaptability, drought-resistance, vertical-stripe, and good-intensity. It is one of the excellent hardwoods and is classified as the national second-class protective tree species in China. *Quercus gilva* is not only a rare timber tree species, but also has ecological functions, such as water conservation, soil and water conservation, disaster prevention and mitigation, etc. It is a promising tree species for afforestation and urban greening (Zhu 2014). In this study, we report the complete chloroplast genome (cp) of *Q. gilva* based on Illumina pair-end sequencing data.

The plant material of *Q. gilva* was collected from Fujian province, China (Shangping Village, Jianou:118°53′33.93″E, 27°31′54.75″N), and dried into silica gel immediately. The voucher specimen is kept at the Herbarium of College of Forestry, Fujian Agriculture and Forestry University (specimen code FAFU0818).

DNA was extracted from fresh leaf tissue, with 500 bp randomly interrupted by the Covaris ultrasonic breaker for library construction. The constructed library was sequenced by PE150 Illumina Hiseq Xten platform, approximately 2GB data was generated. Illumina data were filtered by script in the cluster (default parameter: -L 5, -p 0.5, -N 0.1). The complete plastid genome of *Fagus engleriana* (GeneBank accession: KX852398) was used as reference, plastid genome of *Q. gilva* was assembled by GetOrganelle pipe-line (https://github.com/Kinggerm/GetOrganelle), it can get the plastid-like reads, and the reads were viewed and edited by Bandage (Wick et al. 2015). Assembled choroplast genome annotation was based on the comparison with *F. engleriana* by Geneious v 11.1.5 (Biomatters Ltd., Auckland, New Zealand) (Kearse et al. 2012). The annotation result was drawn with the online tool OGDRAW (http://ogdraw.mpimp-golm.mpg.de/) (Lohse et al. 2013).

The complete plastid genome sequence of *Q. gilva* (GenBank accession: MK986651 ) was 160,763 bp in length, with a large single-copy (LSC) region of 90,292 bp, a small single-copy (SSC) region of 18,831 bp, and a pair of inverted repeats (IR) regions of 25,820 bp. Complete chloroplastid genome contains 128 genes, there were 80 protein-coding genes, 40 tRNA genes, and 8 rRNA genes. The complete genome GC content was 36.9%. In order to reveal the phylogenetic position of *Q. gilva* with other members of Fagaceae, a phylogenetic analysis was performed based on 24 complete chloroplast genomes of Fagaceae, and two taxa (*Alnus cordata*, *Betula cordifolia*) as outgroups. They were all downloaded from NCBI GenBank. The sequences were aligned by MAFFT v7.307 (Katoh and Standley 2013), and the phylogenetic tree was constructed by RAxML (Stamatakis 2014). The phylogenetic tree showed that *Q. gilva* was most closely related to *Q. sichourensis* with strong support (Figure 1).

**Figure 1.**
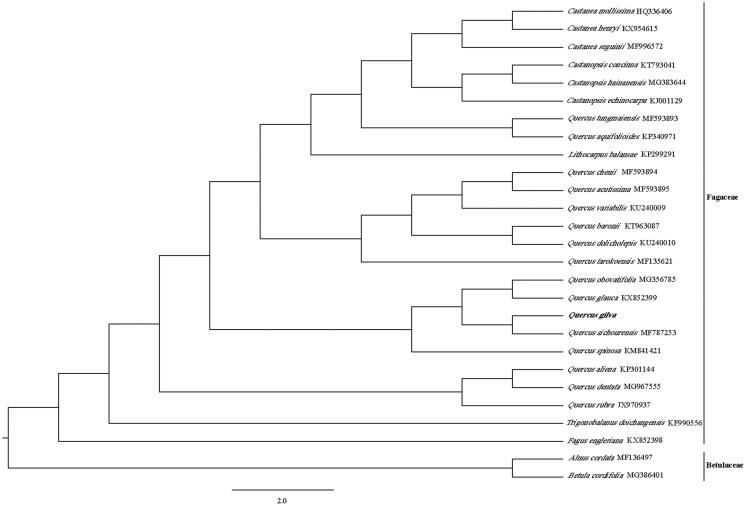
Phylogenetic analysis of 24 species of Fagaceae and two taxa (*Alnus cordata*, *Betula cordifolia*) as outgroup based on plastid genome sequences by RAxML, bootstrap support value near the branch.
